# TG-DSC and TG-FTIR Studies of Annelated Triazinylacetic Acid Ethyl Esters—Potential Anticancer Agents

**DOI:** 10.3390/molecules28041735

**Published:** 2023-02-11

**Authors:** Agnieszka Ostasz, Renata Łyszczek, Krzysztof Sztanke, Małgorzata Sztanke

**Affiliations:** 1 Department of General and Coordination Chemistry and Crystallography, Faculty of Chemistry, Maria Curie-Skłodowska University, M.C. Skłodowskiej Sq. 2, 20-031 Lublin, Poland; 2 Laboratory of Bioorganic Compounds Synthesis and Analysis, Medical University of Lublin, 4A Chodźki Street, 20-093 Lublin, Poland; 3 Department of Medical Chemistry, Medical University of Lublin, 4A Chodźki Street, 20-093 Lublin, Poland

**Keywords:** annelated triazinylacetic acid ethyl esters, anticancer agents, thermal stability, thermal behaviour, thermal degradation course, TG-DSC, TG-FTIR

## Abstract

To avoid problems associated with the storage and processing of newly developed potential medicines, there is a need to carry out thermal studies in the preclinical phase of drug development. The thermal behaviour and decomposition pathway of a whole novel class of patented potential molecular pharmaceutics, i.e., ethyl 2-[4-oxo-8-(R-phenyl)-4,6,7,8-tetrahydroimidazo[2,1-*c*][1,2,4]triazin-3-yl]acetates (**1**–**6**) were reported for the first time in inert and oxidative atmospheres. The experiments were conducted with the use of simultaneous thermogravimetry/differential scanning calorimetry (TG-DSC) and simultaneous thermogravimetry coupled with Fourier transform infrared spectroscopy (TG-FTIR). The decomposition pathways of compounds **1**–**6** were found to be different under oxidative and inert conditions. It was proven that the investigated molecules reveal higher thermal stability under a synthetic air atmosphere than under a nitrogen atmosphere, and their decomposition is preceded by the melting process. Among all the investigated compounds, only the *meta*-chloro derivative (**4**) was found to exhibit interesting polymorphic behaviour at a low heating rate (10 °C min^−1^). It was proven that the oxidative decomposition process of the studied molecules proceeds in three overlapping stages accompanied by strong exothermic effects. Additionally, it was concluded that the title compounds were stable up to a temperature of 195–216 °C in an atmosphere of synthetic air, and their thermal stability decreased in the order of R at the benzene ring: 4-CH_3_ > 3,4-Cl_2_ > 4-Cl > H > 2-OCH_3_ > 3-Cl.

## 1. Introduction

**Figure 1:**
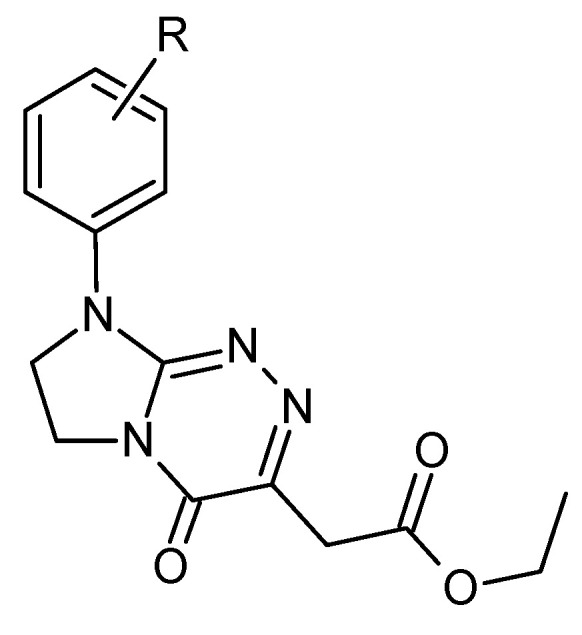
Structures of the studied compounds: **1**. R = H, **2**. R = 4-CH_3_, **3**. R = 2-OCH_3_, **4**. R = 3-Cl, **5**. R = 4-Cl, **6**. R = 3,4-Cl_2_.

Esters are commonly used in medicine because, as less polar compounds than carboxylic acids, they more easily penetrate cell membranes and thus are more bioavailable [1]. Ethyl esters of 2-[4-oxo-8-(R-phenyl)-4,6,7,8-tetrahydroimidazo[2,1-*c*][1,2,4]triazin-3-yl]acetic acid (**1**–**6**) (Figure 1) belong to an important class of heterocyclic compounds with the protected acetic acid function as an acetic acid ethyl ester. The title molecules containing the same privileged scaffold and ester functional group are known due to their disclosed medical application as possible anticancer agents [2]. They are of particular significance in the treatment of human multiple myelomas, both resistant and susceptible to thalidomide, and human tumours of the breast and cervix [2,3]. The most selective and, therefore, the best in this class of small molecules is ethyl 2-[8-(3-chlorophenyl)-4-oxo-4,6,7,8-tetrahydroimidazo[2,1-*c*][1,2,4]triazin-3-yl]acetate (**4**), which is capable of evoking significantly higher necrosis rates in tumour than in normal cells [3]. The investigated compounds have been developed as potential ester prodrugs that should cross cell membranes (including the blood–brain barrier) better than their more polar structural congeners with the acetic acid functional group [4]. An adsorptive stripping voltammetric procedure for the quantitative determination of the most selective anticancer agent (**4**) has been developed and reported as the first analytical method with potential applicability in clinical analytics [5].

Thermal analysis methods are commonly used tools for the characterisation of pharmaceutical raw materials, chemical compounds with potential therapeutic properties and drugs approved for use. Due to the specific application of heterocyclic compounds used in pharmacy, understanding the impact of temperature on their properties is an important research task. Knowledge about the thermal behaviour of molecular pharmaceuticals provides valuable information about the compounds, such as the purity, thermal stability, degree of solvation, decomposition mechanisms or their degradation products. Thermal analysis methods also allow for the study of crystallisation and melting processes, which are crucial for the detection of polymorphic forms of the compound, which, as is known, may exhibit distinct biological activities [6,7,8]. Furthermore, the DSC measurements are employed to study phase change materials [9,10,11] that are widely used in various engineering applications.

The thermolysis, stability in inert and oxidative conditions and decomposition products of structurally diverse and pharmaceutically important classes of molecules bearing the ester functional group/groups have been previously investigated and disclosed [12,13,14,15,16]. In addition, the thermal decomposition mechanism in an important class of potential anticancer agents, i.e., diethyl butanedioates bearing the imidazolidine template, has been previously explained [12].

However, none of these papers concerned molecules such as our patented, published and potentially useful in clinical analytics ethyl 2-[4-oxo-8-(R-phenyl)-4,6,7,8-tetrahydroimidazo[2,1-*c*][1,2,4]triazin-3-yl]acetates (**1**–**6**) [2,3,4,5], whose thermal behaviour is described for the first time in detail in the current paper. As the thermal properties and thermal degradation mechanism of the title class of possible new anticancer agents with prospective medical use (**1**–**6**) are unknown, the main goal of the present investigation is to determine their thermal stability as well as thermal behaviour under oxidative and inert atmosphere conditions. This article focuses on the thermostability determination of candidates for medical applications belonging to a novel class of functionalised heterocycles by investigating their thermal degradation pathways and tries to explain the relationships between the structure and thermal stability of the analysed compounds. This paper also describes the thermal degradation mode of the title compounds, showing the degradation products (including gaseous ones). Therefore, the obtained knowledge about the range of thermal stability and thermal properties of the molecules recruited is important and will be helpful in a further evaluation of these possible pharmaceutics that are in the preclinical phase of drug development.

## 2. Results and Discussion

The structures of the investigated molecules **1**–**6** are given in Figure 1. The thermal behaviour of all the compounds was determined in oxidative (an air atmosphere) as well as in inert (a nitrogen atmosphere) conditions. The thermal properties of these patented drug candidates are presented for the first time. The results presented in this study are essential for the further characterisation of this new class of potential pharmaceutics.

### 2.1. Thermal Behaviours of Compounds ***1**–**6*** in an Oxidative Atmosphere

**Figure 2:**
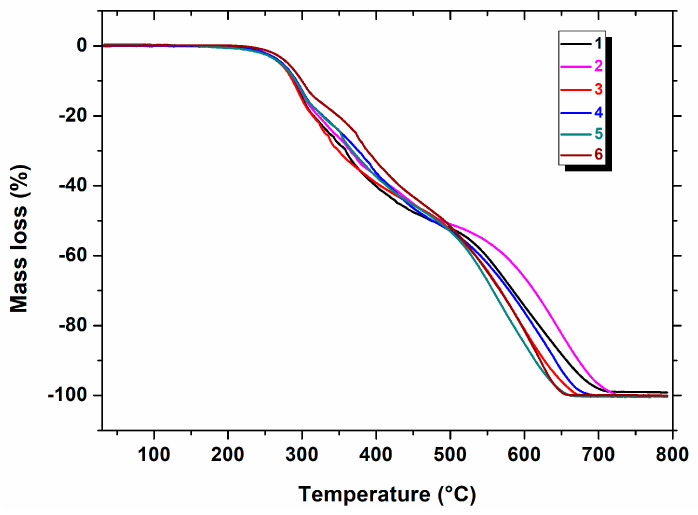
TG curves for compounds **1**–**6** (an air atmosphere).

The simultaneous thermogravimetry (TG) and differential scanning calorimetry (DSC) methods were applied for the determination of the thermal properties of the investigated heterocyclic esters (**1**–**6**) in an oxidative atmosphere. It was found that all the compounds are thermally stable under an air atmosphere, and their thermal stability increases in the order: **4** (195 °C) < **3** (197 °C) < **1** (200 °C) < **5** (206 °C) < **6** (211 °C) < **2** (216 °C), taking into account their mass changes during heating (Figure 2 and Table 1). This thermal property would be very important in the case of approval of the investigated molecules as pharmaceutics. It was proven that for medicines stable at temperatures much higher than ambient temperature, changes in storage temperature in the range from 20 °C to 45 °C have practically no impact on their shelf life [17,18].

The melting enthalpy (ΔH_m)_ values received in oxidative conditions are in the range from 6.75 kJ mol^−1^ to 29.90 kJ mol^−1^ (Table 1). The highest ΔH_m_ value was seen for compound **3** containing the 2-OCH_3_ substituent at the benzene ring. Additionally, it was found that the replacement of a hydrogen atom at the benzene ring by the 4-CH_3_ group or by one or two chlorine atom/atoms leads to a decrease in the ΔH_m_ values. This means that less heat energy is required to convert a mole of a solid at its melting point into a liquid without an increase in temperature for compounds **2**, **4**, **5** and **6** as compared to the heat energy evaluated for the parent compound (**1**).

At higher temperatures, three main stages of decomposition could be distinguished on the TG curves (Figure 2). The first mass loss of 16.04–23.70% for all the compounds (**1**–**6**) was recorded in the first stage of their decomposition in the temperature range of 195–339 °C. All the resulting intermediates were unstable and underwent further decomposition processes. The second stage of decomposition took place in the temperature range of 315–502 °C. The corresponding mass losses were slightly higher at 23.13–27.48%. The last step of compounds’ degradation occurred at a temperature above 448 °C, and it was accompanied by significant mass losses in the range of 48.71–56.65%. The final decomposition temperature, where a plateau on the TG curve occurred, was in the range of 671–765 °C (Table 1). From the above data, it is evident that compound **2** is the most thermally stable, as its molecule degrades at the highest temperature.

**Figure 3:**
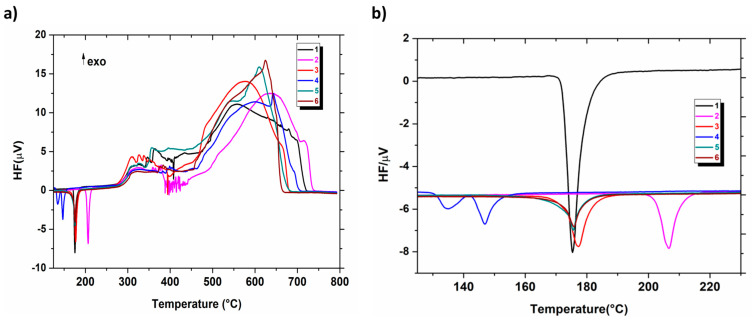
DSC curves for compounds **1**–**6** (an air atmosphere) (**a**) in the temperature range of 30–800 °C and (**b**) in the temperature range of 125–230 °C. HF – heat flow.

DSC analysis was performed simultaneously with the recording of TG curves. The results obtained from the application of this method allowed for a better understanding of the temperature influence on the physicochemical properties of the investigated compounds as well as the mechanism of their decomposition. Heating the compounds resulted in the melting process, which takes place before their degradation. The DSC curves recorded in the temperature range of 125–230 °C showed one sharp endothermic peak for compounds **1**–**3** and **5**–**6**, while two endothermic effects for compound **4** were noticed (Figure 3). The previous ^1^H NMR, ^13^C NMR, HPLC and electrochemical investigations [2,3,4,5] confirm that compound **4** is a homogeneous substance of high purity, so the attendance of the two peaks on the DSC curve cannot be due to its contamination.

**Figure 4:**
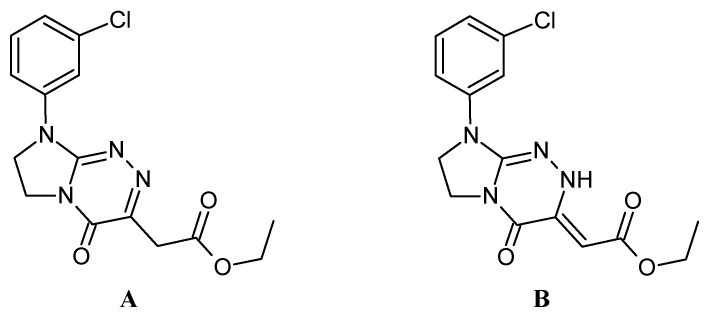
The structurally distinct forms of the compound **4**: ethyl 2-[8-(3-chlorophenyl)-4-oxo-4,6,7,8-tetrahydroimidazo[2,1-*c*][1,2,4]trazin-3-yl]acetate—the ketimine polymorph (**A**) and ethyl 2-[8-(3-chlorophenyl)-4-oxo-2,6,7,8-tetrahydroimidazo[2,1-*c*][1,2,4]trazin-3(4*H*)-ylidene]acetate—the enamine polymorph (**B**).

The fact that the solid–liquid (melting) phase transition for the majority of tested compounds (**1**–**3** and **5**–**6**) was described by one sharp DSC peak testifies to their high purity. This is a favourable thermal property, and these anticancer active compounds are expected to have higher physical and chemical stability compared to amorphous substances. The parent structure **1**—which does not contain a substituent attached to the phenyl moiety—exhibits the melting temperature (T_peak_) at 175 °C. Replacing the hydrogen atom at the benzene ring by a methyl group, methoxy substituent or one/two chlorine atom/atoms causes an increase in the melting temperature (except in the case of compound **4**). The highest melting point occurs for molecule **2** with an electron-donating methyl group in the *para* position (T_peak_ = 206 °C). For molecules such as compound **3** (the *ortho*-OCH_3_ derivative), **5** (the *para*-Cl derivative) and **6** (the *meta, para*-Cl_2_ derivative), the melting point increase is slight (by 1 and 2 °C) compared to parent structure **1** (Table 1). An interesting melting behaviour was observed for compound **4**. The DSC curve of this compound exhibits two endothermic effects (T_peak_ = 135 °C and 147 °C, the second of which is more acute), as can be seen in Figure 3b. They are probably related to the two polymorphic forms (A and B) of the *meta*-Cl derivative, which are shown in Figure 4. The heat of the effect observed at a lower temperature is 6.75 kJ·mol^−1^, while the second effect has a higher energy of 11.36 kJ·mol^−1^. The type of solvent, the rate of crystallisation, the presence of other substances, the mixing and the concentration of the reagents influence the resulting polymorphic form. Changing one of these factors can lead to the transformation of polymorphs. Usually, the most stable form has the highest melting point. In both crystal phases of compound **4**, visible in the DSC curves, it can be stated that they have a different arrangement and different conformation of the molecules in the crystal but have no solvent molecules in their structure. The interesting melting phenomenon, which has already been reported for a number of molecules [19,20], most likely results from the possibility of solid phase proton transfer in the molecule of **4** and consequently leads to its polymorphic transformation at a specific heating rate in result of the bond change (i.e., desmotropism). It is most likely that compound **4**, i.e., more precisely, its proven ketimine structure A, is transformed into its more stable enamine polymorph B (whose structure can be stabilised by an intramolecular hydrogen bond between the ester C=O and NH group of the triazine ring) [21]. It seems that the type of solvent used for crystallisation (DMF) and the rate of crystallisation favour the polymorphic behaviour of **4**. No polymorphism was observed in the case of the remaining molecules.

As it results from the profiles of the DSC curves, the melting process of compounds **1**–**6** is followed by the evaporation processes and/or decomposition of molten compounds. The DSC curves show several overlapping peaks that are very difficult to assign due to the complexity of the processes involved. They are probably related to the processes of thermal dissociation of chemical bonds. Above 450 °C, on the DSC curves, the significant exothermic effects appeared as the result of the combustion processes of solid unstable intermediates.

### 2.2. TG-FTIR Analysis of Compounds ***1**–**6*** in a Nitrogen Atmosphere

**Figure 5:**
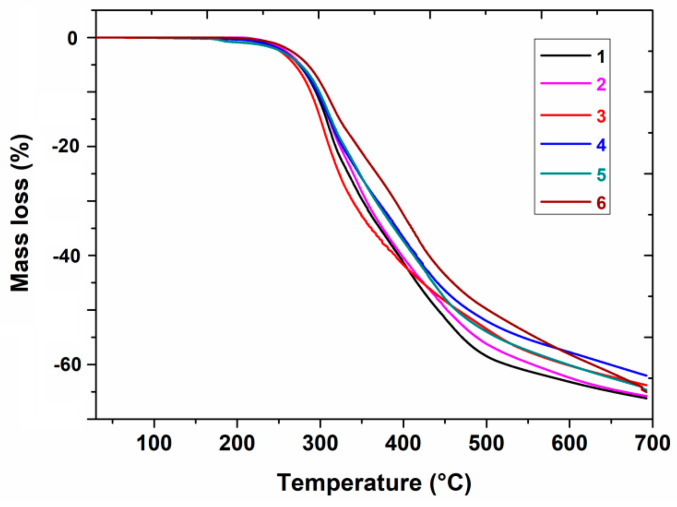
TG curves of compounds **1**–**6** recorded in a nitrogen atmosphere.

Coupling of the thermogravimetric analysis (TG) and a Fourier transform infrared spectroscopy (FTIR) method was applied for the determination of the thermal stability and decomposition mechanism of the investigated compounds (**1**–**6**) in a nitrogen atmosphere as well as the identification of evolved volatile products of their degradation. It was found that all the tested compounds (**1**–**6**) are thermally stable materials under inert conditions and their thermal stability can be ordered as follows: **4** (119 °C) < **5** (157 °C) < **6** (177 °C) < **3** (178 °C) < **1** (184 °C) < **2** (201 °C) based on the analysis of their thermogravimetric curves (Figure 5).

Comparing the temperature of decomposition beginning in the corresponding compounds in nitrogen and air, obviously an impact of the atmosphere heating on the compound stability is seen. At first, a decrease in the thermal stability of all compounds is noticed. The fact that this influence has a double character is quite surprising. Taking into account the thermal stability of parent compound (**1**), introducing the substituents such as methyl, methoxy and chlorine groups at the benzene ring in different positions strongly influences the stability of the explored compounds. Compound **2**, which exhibits the highest thermal stability, is structurally characterised by the presence of a methyl substituent in the *para* position at the benzene ring. On the other hand, replacing the hydrogen atom of the benzene ring with a chlorine atom in the *meta* position, as took place in compound **4**, leads to a drastic decrease in its stability under inert conditions but a slight decrease in its stability under oxidative conditions. An impact of the methoxy group in an *ortho* position as well as two chlorine substituents in positions 3 and 4 of the phenyl moiety is very similar. In both cases, a decrease in compound stability in relation to parent structure (**1**) is noticed. These observations do not resonate with those previously reported for thiophenylated fused triazinones [22]. In this class of compounds, the substitution of hydrogen atoms in positions 4 or 3 and 4 through one or two chlorine atoms, respectively, resulted in an increase in thermal stability compared to parent structure (**1**). The similar influence of two chlorine substituents at the benzene ring on the thermal stability of the trifluoromethylated fused triazinones was also observed [23]. Presumably, these observations can be explained in terms of the overall geometry of the molecules, their relative position and packing as well as their molecular interactions.

A detailed analysis of the TG curves of the tested compounds suggests that their decomposition occurs in overlapping stages without forming thermally stable solid products. Compared to the TG curves recorded in the air, it is impossible to separate these stages. Their shapes are dominated by one significant mass loss of 66.39–64.01% in the temperature range of 119–700 °C. The mass of solid residues composed of unburnt carbon increased as follows: 33.61% (**1**), 34.72% (**2**), 35.80% (**6**), 35.89% (**3** and **5**) and 35.99% (**4**).

**Figure 6:**
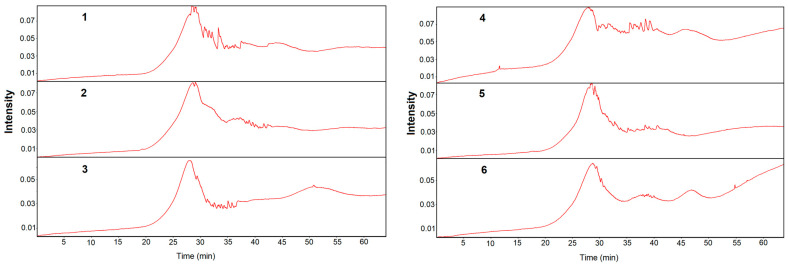
Gram–Schmidt plots of compounds **1**–**6** in nitrogen.

Besides the TG curves, Gram–Schmidt plots as well as FTIR spectra of volatile products of their degradation point out to their multistep pyrolysis process. The Gram–Schmidt plots showing the intensity of the evolved gases during the heating of the compounds, which are shown in Figure 6, also confirm their different ways of degradation.

The highest intensity of the emitted gases during pyrolysis of compounds **1**–**6** is observed between 25 and 31 min of heating, which corresponds to a temperature range of about 270–340 °C (Figure 6). At higher temperatures, very complex processes of degradation occur as evidenced by the multipeak profiles of the Gram–Schmidt plots.

**Figure 7:**
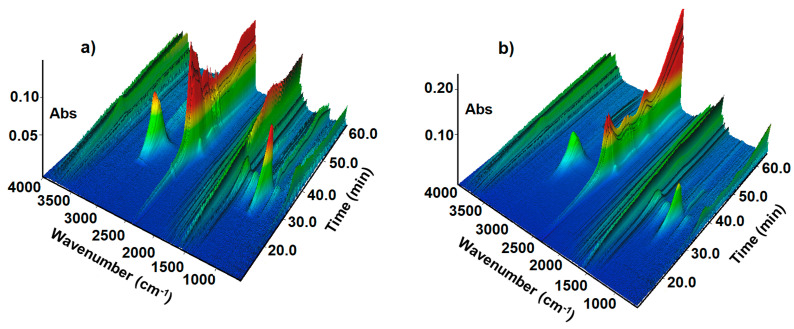
FTIR spectra of volatile products of decomposition in nitrogen for (**a**) compound **2** and (**b**) compound **6**.

The 3D presentations of the FTIR spectra of the evolved gases during the heating of compounds **2** and **6** as two representatives are given in Figure 7.

**Figure 8:**
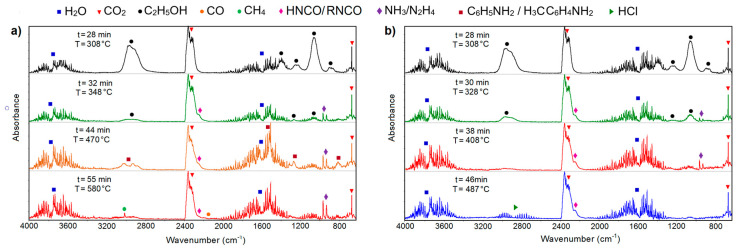
FTIR spectra that were recorded at different temperatures in a nitrogen atmosphere for (**a**) compound **2** and (**b**) compound **6**.

The first gaseous species recognised on the FTIR spectra as products of all compounds’ decomposition were ethyl alcohol, water and carbon dioxide (Figure 7 and Figure 8). The presence of such compounds can be explained as the result of the cleavage of the bond between ethyl ester of acetic acid moiety and 1,2,4-triazine skeleton. The FTIR spectra display several very characteristic bands for the released C_2_H_5_OH molecules. The split band in the region of 3100–2800 cm^−1^ with maxima at 2965 cm^−1^ and 2913 cm^−1^ was assigned to the asymmetric and symmetric stretching vibrations of the CH_3_ group. The strong band located at 1057 cm^−1^ is characteristic of the C–O stretching vibrations of the primary alcohol, while those at 897 cm^−1^ were assigned to the stretching vibrations of the CCO group [24]. The evolved carbon dioxide molecules give rise to the characteristic bands at 2358, 2322 and 697 cm^−1^ from their stretching and deformation vibrations. Simultaneously, the bands in the regions of 4000–3200 cm^−1^ and 1800–1300 cm^−1^ were recorded due to the stretching and deformation vibrations of water molecules (Figure 8) [25,26].

At higher temperatures, further decomposition of the solid residues took place. The FTIR spectra recorded above 29 min (328 °C) of heating exhibit very diagnostic double bands with maxima at 966 and 930 cm^−1^ derived from the asymmetric and symmetric stretching vibrations of NH groups from ammonia or hydrazine [27,28]. Along with these molecules, isocyanic acid (HNCO) and/or its derivatives (RNCO) are also evolved. This conclusion was driven based on the broadening of the multipeak band observed in the range of 2200–2100 cm^−1^ [29]. Deep analysis of such a band allows for distinguishing the band at 2251 cm^−1^ as characteristic for the stretching vibrations of NCO moieties. Simultaneously, the FTIR spectra display diagnostic bands at 965 and 930 cm^−1^ due to the evolution of ammonia and/or hydrazine molecules. These compounds can be regarded as products of the cleavage of triazine moieties. An increase in the temperature leads to the release of aniline and *p*-toluidine molecules [30]. The FTIR spectra show bands in the range of 3150–3000 cm^−1^ derived from the stretching vibrations of the CH groups. The most distinctive bands for these compounds are observed at 1270 and 754 cm^−1^ due to the stretching vibrations of the CN group and wagging vibrations of NH, respectively. The liberation of such compounds is especially observed during decomposition of the compounds **1**, **2** and **5** (Figure 8a). The FTIR spectra reflect also the presence of substituents in the benzene ring. Among the volatile products of decomposition of compound **2**, methane and carbon monoxide are also clearly observed. The stretching vibrations of the CH bonds from the methane molecules give rise to the characteristic group of bands in the range of 3200–2900 cm^−1^ with a maximum of 3017 cm^−1^. A very weak double band in the range of 2200–2000 cm^−1^ confirms the evolution of carbon monoxide [26]. The FTIR spectra of compound **6**, which contains in its structure two chlorine atoms, show split bands in the range of 3100–2600 cm^−1^ as a result of the evolution of the HCl molecules [31] (Figure 8b).

### 2.3. Assessment of the Risk of Side Effects and the Impact on Red Blood Cells of the Investigated Compounds (***1**–**6***)

Taking into account the fact that the title compounds, as anticancer drug candidates with potential therapeutic use [2,3], are characterised by high thermal stability (described in this paper) and optimal pharmacokinetic properties [4], the determination of their safety/toxicity profile is justified.

In the preclinical phase of drug development, it is important to assess the risk of serious adverse effects of new drug candidates. The molecules revealing mutagenic, tumorigenic, irritating and reproductive effects should not be tested on living organisms. Therefore, a useful in silico tool—the OSIRIS Property Explorer (available online at http://www.organic-chemistry.org/prog/peo/; accessed on 3 January 2023)—was applied to predict the possibility of the appearance of adverse side effects in this novel class of compounds. The adverse side effects risk predictor locates all structural fragments that give rise to toxicity alerts if they are present in the molecule investigated. A set of these fragments was taken from the Registry of Toxic Effects of Chemical Substances database. This database contains a large number (about 20,000) of chemical substances revealing mutagenic, tumorigenic, irritant and reproductive effects and medicines as a control group. The prediction result indicates no risk (score 1.0; green colour), medium risk (score 0.8, yellow colour) and high risk (score 0.6; red colour) of the undesired effect. In the case of our annelated triazinylacetic acid ethyl esters (**1**–**6**), according to the OSIRIS programme, none of the investigated compounds poses a risk of mutagenic, carcinogenic, irritating or reproductive effects (Table S1 in the Supplementary material). This was to be expected, as no genotoxophore fragments are present in the molecules of the compounds studied.

The evaluation of the toxicity profile of pharmacologically relevant compounds is an important task in the preclinical phase of drug development. Therefore, the effect of compounds **1**–**6** on the most numerous cells in the living organism, i.e., erythrocytes, was evaluated in an ex vivo model. It was found that all annelated triazinylacetic acid ethyl esters after incubation with red blood cells do not cause any haemolytic effects, and thus they are safe for erythrocytes (Table S2 in the Supplementary material). This confirms the very good safety profile of these compounds, which is particularly important due to their potential usefulness in cancer therapy. On the other hand, the antihaemolytic properties of compounds **1**–**6** were evaluated ex vivo on red blood cells exposed to reactive oxygen species, such as peroxyl radicals or hydrogen peroxides, which, by inducing oxidation of membrane proteins and lipids, lead to erythrocyte membrane damage and ultimately to haemolysis. The ability of annelated triazinylacetic acid ethyl esters to inhibit oxidative hemolysis, and thus to protect erythrocytes against oxidative damage, was compared to that of ascorbic acid or trolox (Figure S1A,B in the Supplementary material). It was proven that all the tested molecules protect the red blood cells from oxidative damage. This effect was dependent on the structure of the compound. The most effective in inhibiting AAPH-induced haemolysis were found to be compounds **3** and **4**, whose antihaemolytic activity was 88% and 85% of ascorbic acid activity, respectively. In turn, compounds **3** and **2** inhibited the most effectively H_2_O_2_-induced hemolysis, and their activity was 89% and 86%, respectively, compared to trolox. The remaining annelated triazinylacetic acid ethyl esters also protected erythrocytes against oxidative haemolysis, and their activity ranged from 61% to 79% in relation to the activity of antioxidant standards.

Both the lack of risk of side effects and the beneficial impact on erythrocytes of the title compounds (**1**–**6**) make them safe drug candidates suitable for further research.

## 3. Materials and Methods

### 3.1. Short Description of the Investigated Compounds (***1**–**6***)

Ethyl 2-[4-oxo-8-(R-phenyl)-4,6,7,8-tetrahydroimidazo[2,1-*c*][1,2,4]triazin-3-yl]acetates (**1**–**6**) belonging to fused azaisocytosine congeners have been synthesised for the purposes of thermal studies according to efficient synthetic approaches previously patented and published [2,3]. The structures of molecules **1**–**6** have been confirmed by ^1^H-NMR/^13^C-NMR spectra and elemental analysis, and established on the basis of the performed ^13^C, ^1^H HMBC and HMQC correlations for the ethyl ester of 2-(4-oxo-8-phenyl-4,6,7,8-tetrahydroimidazo[2,1-*c*][1,2,4]triazin-3-yl)acetic acid (**1**) [3]. The purity and homogeneity of all the compounds intended for thermal studies (**1**–**6**) have been previously evaluated under reaction and the purification conditions employed. All these ones have been obtained and described [3] as homogenous, pure, crystalline solids with sharp melting points and microanalyses within ±0.4 percent of the calculated values. They have been reported to reveal not only enhanced anticancer effects in malignant human multiple myeloma cells (MM1R, MM1S) but also antiproliferative activities against human tumours of the breast (T47D) and cervix (HeLa) [3]. In addition, their mode of anticancer action and very low toxicities towards normal human skin fibroblasts have been previously documented [3].

### 3.2. Thermal Analysis Methods

The TG-DSC curves were recorded using the SETSYS 16/18 thermal analyser (Setaram, Caluire, France) in an air atmosphere. The samples (mass of 5–8 mg) were heated in open alumina cylindrical (100 μL) crucibles from 30 to 800 °C with a heating rate of 10 °C min^−1^ (airflow 12.5 cm^3^ min^−1^). Simultaneous TG-FTIR analyses were carried out on the Q5000 (TA Instruments, New Castle, DE, USA) analyser coupled with a Nicolet 6700 (Thermo Scientific, Waltham, MA, USA) spectrophotometer. The samples of about 30 mg were heated from room temperature up to 700 °C at a heating rate of 10 °C min^−1^ (nitrogen flow 25 cm^3^ min^−1^) in open plate platinum crucibles. The IR cell maintained at 250 °C was connected online to the Q5000 thermal analyser (TA Instruments, New Castle, DE, USA) using stainless steel heated to 240 °C. The identification of volatile decomposition products of compounds **1**–**6** was made using the database OmnicSpecta 2.0 software.

## 4. Conclusions

The thermal stability and thermal properties of ethyl 2-[4-oxo-8-(R-phenyl)-4,6,7,8-tetrahydroimidazo[2,1-*c*][1,2,4]triazin-3-yl]acetates (**1**–**6**)—regarded as potential anticancer agents—were determined in oxidative and inert conditions. They are thermally stable at about 200 °C in an oxidative atmosphere. Therefore, if they are registered as pharmaceuticals, there will be no problems with their storage at room temperature as well as processing by the pharmaceutical industry. All the investigated compounds reveal higher thermal stability under oxidative conditions than under inert conditions. Therefore, it can be assumed that oxygen enhances the decomposition activation energy by acting as an inhibitor of the decomposition process of the studied polynitrogenated small molecules. The decomposition of the studied molecules is preceded by their melting process. A single endothermic effect observed on the DSC curves of compounds **1**–**3** and **5**–**6**, assigned to the melting process, confirms their high purity. The advantage of these heterocyclic esters is that they do not undergo any polymorphic transformations when studied at a low heating rate. On the other hand, the appearance of two endothermic effects for compound **4** suggests the presence of its two polymorphs and the thermal transformation of a less stable crystal form to a more stable crystal form. The decomposition of the investigated compounds occurs in three overlapping stages accompanied by strong exothermic effects above 450 °C. However, in a nitrogen atmosphere, their thermal stability, as well as decomposition pathways, were different. Analysis of the FTIR spectra recorded during the heating of the investigated compounds (**1**–**6**) enabled us to distinguish the main three stages of their decomposition related to the defragmentation of the investigated molecules during their heating in nitrogen. In the first decomposition stage, the FTIR spectra show bands from water, carbon dioxide and ethyl alcohol molecules as the defragmentation products of the acetic acid ethyl ester moiety. The attendance of ammonia and/or hydrazine along with isocyanic acid and its derivatives primarily occurred in the second decomposition stage. At last, in the third stage of their decomposition, aniline and its derivatives are released.

## Figures and Tables

**Table 1:** Thermal data for compounds **1**–**6** (an air atmosphere).

Sample	Melting Process			Decomposition Process					
	T_onset_ [°C]	T_peak_ [°C]	ΔH_m_ [kJ·mol^−1^]	Step 1		Step 2		Step 3	
				ΔT_1_ [°C]	Δm_1_ [%]	ΔT_2_ [°C]	Δm_2_ [%]	ΔT_3_ [°C]	Δm_3_ [%]
**1**	172	175	26.31	200–328	23.26	328–484	27.33	484–718	48.71
**2**	202	206	24.87	216–338	23.70	338–502	27.48	502–765	48.72
**3**	172	177	29.90	197–315	20.55	315–454	25.33	454–692	54.09
**4**	131 143	135 147	6.75 11.36	195–336	21.83	336–476	28.25	476–705	49.87
**5**	172	176	25.21	206–339	22.57	339–448	23.13	448–680	54.18
**6**	171	176	19.79	211–324	16.04	324–450	27.22	450–671	56.65

T_onset_—onset temperature of endothermic effect; T_peak_—melting peak temperature; ΔH_m_—melting enthalpy; ΔT_1_, ΔT_2_, ΔT_3_—temperature ranges of decomposition stages; Δm_1_, Δm_2_, Δm_3_—mass losses.

## Data Availability

The data presented in this study are available on request from the authors.

## Supplementary Materials

The following supporting information can be downloaded at: https://www.mdpi.com/article/10.3390/molecules28041735/s1. Table S1. Risk assessment of adverse side effects by OSIRIS Property Explorer for the investigated compounds (**1**–**6**). Table S2. Haemolytic activity of the investigated compounds (**1**–**6**) at a 0.15 mM concentration. Figure S1. (**A**) Antihaemolytic activities (in the model of rat erythrocytes exposed to AAPH) of compounds **1**–**6** in relation to ascorbic acid. (**B**) Antihaemolytic activities (in the model of rat erythrocytes exposed to H_2_O_2_) of compounds **1**–**6** in relation to trolox.

## Sample Availability

Samples of the investigated compounds are available from the authors.
