# Supplementary material for: TG-DSC and TG-FTIR Studies of Annelated Triazinylacetic Acid Ethyl Esters—Potential Anticancer Agents
Source: Molecules. 2023 Feb 11;28(4):1735. doi: 10.3390/molecules28041735 (PMC9967103; doi:10.3390/molecules28041735)
Supplement: Supplementary file 1 [file molecules-28-01735-s001.zip › molecules-2188000-supplementary.pdf]

molecules

MDPI

Supplementary material

TG-DSC and TG-FTIR Studies of Annelated Triazinylacetic Acid Ethyl Esters—Potential Anticancer Agents

Agnieszka Ostasz [1], Renata Łyszczek [1], Krzysztof Sztanke [2,*] and Małgorzata Sztanke [3]

[1] Department of General and Coordination Chemistry and Crystallography, Faculty of Chemistry, Maria Curie-Skłodowska University, M.C. Skłodowskiej Sq. 2, 20-031 Lublin, Poland
[2] Laboratory of Bioorganic Compounds Synthesis and Analysis, Medical University of Lublin, 4A Chodźki Street, 20-093 Lublin, Poland
[3] Department of Medical Chemistry, Medical University of Lublin, 4A Chodźki Street, 20-093 Lublin, Poland
* Correspondence: krzysztof.sztanke@umlub.pl

Table S1. Risk assessment of adverse side effects by OSIRIS Property Explorer for the investigated compounds (**1**–**6**).

Compound	Mutagenicity	Tumorigenicity	Irritating effects	Reproductive effects
**1**				
**2**				
**3**				
**4**				
**5**				
**6**				

– no risk, score: 1.0.

Table S2. Haemolytic activity of the investigated compounds (**1-6**) at a 0.15 mM concentration.

Compound / control		Haemolytic activity (in %)
Compound	**1**	0
	**2**	0
	**3**	0
	**4**	0
	**5**	0
	**6**	0
A positive control	10% solution of Triton X-100	100
A negative control	Phosphate Buffered Saline	0

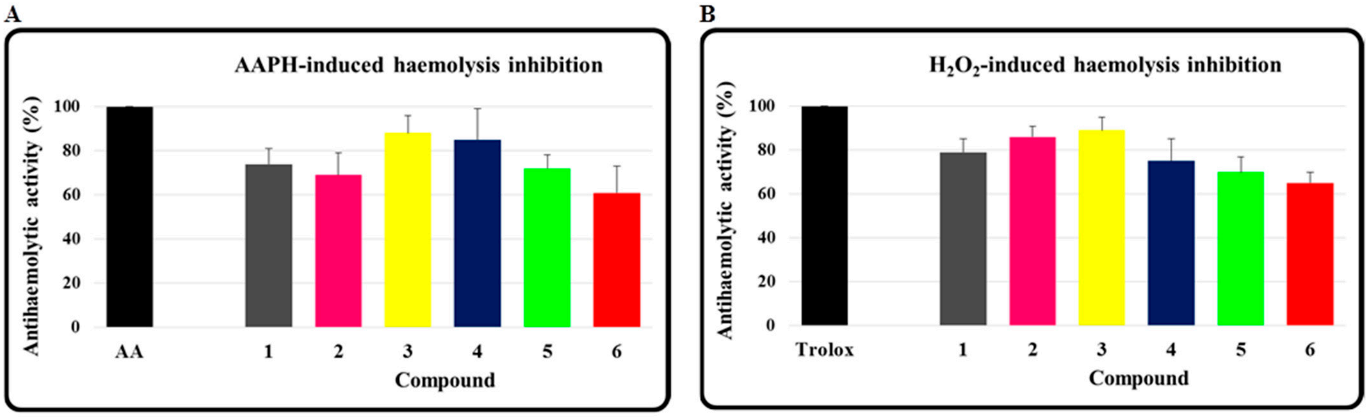

Figure S1. A. Antihaemolytic activities (in the model of rat erythrocytes exposed to AAPH) of compounds **1-6** in relation to ascorbic acid. **B.** Antihaemolytic activities (in the model of rat erythrocytes exposed to H_2O_2) of compounds **1-6** in relation to trolox.
Compounds and standard antioxidants were tested at a concentration of 0.15 mM. AAPH – 2,2′-azobis(2-methylpropionamidine)dihydrochloride; AA – ascorbic acid; H_2O_2 – hydrogen peroxide; trolox – 6-hydroxy-2,5,7,8-tetramethylchroman-2-carboxylic acid. Data (from three independent experiments) are shown as the mean ± standard deviation.

S3. Materials and Methods

S3.1. Haemolysis assays

The haemolytic and antihaemolytic properties of the investigated compounds (**1-6**) were *ex vivo* assessed on erythrocytes of the rat (male Wistar rat; 8-9 weeks old; 200-250 g; the Experimental Medicine Centre, Medical University of Lublin, Poland). After the blood was centrifuged (1500 rpm; 10 min; 4°C), the plasma was separated and the red blood cells were washed three times with phosphate-buffered saline (PBS; pH 7.4; Biomed, Lublin, Poland). A 4% suspension of erythrocytes in PBS was used for further studies.

To evaluate the haemolytic activity of all annelated triazinylacetic acid ethyl esters (**1-6**), each compound at a concentration of 0.15 mM was incubated (37°C; 60 min) with the erythrocyte suspension. Then, the samples were centrifuged (3000 rpm; 10 min) and the absorbances at λ_{max} = 540 nm were measured on a Hitachi U2800 spectrophotometer (Hitachi, Tokyo, Japan). The red blood cells suspension in Triton X-100 (Sigma-Aldrich, Saint Louis, MO, USA) was the positive control (complete haemolysis), while the erythrocyte suspension in PBS was the negative control (no haemolysis).

To assess the antihaemolytic activity of all annelated triazinylacetic acid ethyl esters (**1-6**), each compound at a concentration of 0.15 mM was incubated (37°C; 60 min) with the red blood cells suspension. Then, 40 mM AAPH (2,2'-azobis(2-amidinopropane) dihydrochloride; Sigma-Aldrich, Saint Louis, MO, USA) or 75 mM H_2O_2 (hydrogen peroxide; Sigma-Aldrich, Saint Louis, MO, USA) ice-cold solutions in PBS was added to each sample. After incubation (37°C; 210 or 180 min, respectively), the samples were centrifuged (1000 rpm; 5 min) and the absorbances were measured spectrophotometrically at λ_{max} = 524 or 540 nm in the case of samples containing AAPH or H_2O_2, respectively. The antihaemolytic activity of the tested compounds was calculated in relation to ascorbic acid or trolox.
